# Glycerophospholipids protect stallion spermatozoa from oxidative damage *in vitro*

**DOI:** 10.1530/RAF-21-0028

**Published:** 2021-07-21

**Authors:** Ashlee J Medica, Robert J Aitken, Garth L Nicolson, Alecia R Sheridan, Aleona Swegen, Geoffry N De Iuliis, Zamira Gibb

**Affiliations:** 1 Priority Research Centre for Reproductive Science, College of Engineering, Science and Environmental, and Faculty of Health and Medicine, University of Newcastle, Callaghan, New South Wales, Australia; 2 Institute for Molecular Medicine, Huntington Beach, California, USA

**Keywords:** stallion spermatozoa, sperm membrane, membrane lipid replacement, reactive oxygen species

## Abstract

Stallion sperm membranes comprise a high proportion of polyunsaturated fatty acids, making stallion spermatozoa especially vulnerable to peroxidative damage from reactive oxygen species generated as a by-product of cell metabolism. Membrane lipid replacement therapy with glycerophospholipid (GPL) mixtures has been shown to reduce oxidative damage *in vitro* and *in vivo*. The aims of this study were to test the effects of a commercial preparation of GPL, NTFactor^®^ Lipids, on stallion spermatozoa under oxidative stress. When oxidative damage was induced by the addition of arachidonic acid to stallion spermatozoa, the subsequent addition of GPL reduced the percentage of 4-hydroxynonenal (4-HNE; a key end product of lipid peroxidation) positive cells (32.9 ± 2.7 vs 20.9 ± 2.3%; *P* ≤ 0.05) and increased the concentration of 4-HNE within the spent media (0.026 ± 0.003 vs 0.039 ± 0.004 µg/mL; *P* ≤ 0.001), suggesting that oxidized lipids had been replaced by exogenous GPL. Lipid replacement improved several motility parameters (total motility: 2.0 ± 1.0 vs 68.8 ± 2.9%; progressive motility: 0 ± 0 vs 19.3 ± 2.6%; straight line velocity: 9.5 ± 2.1 vs 50.9 ± 4.1 µm/s; curvilinear velocity: 40.8 ± 10 vs 160.7 ± 7.8 µm/s; average path velocity: 13.4 ± 2.9 vs 81.9 ± 5.9 µm/s; *P* ≤ 0.001), sperm viability (13.5 ± 2.9 vs 80.2 ± 1.6%; *P* ≤ 0.001) and reduced mitochondrial ROS generation (98.2 ± 0.6 vs 74.8 ± 6.1%; *P* ≤ 0.001). Supplementation with GPL during 17°C *in vitro* sperm storage over 72 h improved sperm viability (66.4 ± 2.6 vs 78.1 ± 2.9%; *P* ≤ 0.01) and total motility (53 ± 5.6 vs 66.3 ± 3.5%; *P* ≤ 0.05). It is concluded that incubation of stallion spermatozoa with sub-µm-sized GPL micelles results in the incorporation of exogenous GPL into sperm membranes, diminishing lipid peroxidation and improving sperm quality *in vitro*.

**Lay summary:**

Sperm collection and storage is an important step in many artificial insemination and *in vitro* fertilization regimes for several species, including humans and horses. The sperm membrane, which acts as a protective outer barrier, is made up of fatty acid-containing molecules – called phospholipids. These phospholipids may become damaged by waste products generated by the cell, such as hydrogen peroxide, during non-chilled sperm storage. We aimed to determine if sperm cells were able to repair this membrane damage by supplementing them with phospholipids during non-chilled storage. Sperm was collected from five miniature stallions by artificial vagina, and then supplemented with phospholipids during 72 h sperm storage at 17°C. Our studies show that when stallion sperm are supplemented with phospholipids *in vitro*, they are able to remove their damaged membrane phospholipids and swap them for undamaged ones, aiding in resistance to cellular waste and improving cell health and potential fertility.

## Introduction

The sperm plasma membrane is far more than just an inert barrier. It actively protects spermatozoa against extracellular damage and plays a dynamic role throughout sperm capacitation and in sperm-oocyte cross talk during fertilisation (Flesch & Gadella 2000, Ortega Ferrusola *et al.* 2010). The plasma membrane and additional inner organelle membranes are comprised of several types of phospholipids, each exhibiting a specific and unique structure and distinctive fatty acid profile (Lenzi *et al.* 1996, Fahy *et al.* 2005). These phospholipid classes include those with saturated, mono-unsaturated and polyunsaturated fatty acids (PUFA); sperm membranes are comprised of an extremely high percentage of the latter (22:6; polyunsaturated-to-saturated; Aitken *et al.* 1989). This high proportion of PUFA makes spermatozoa especially vulnerable to peroxidative damage from reactive oxygen species (ROS) such as superoxide, and hydrogen peroxide (H_2_O_2_; Saleh & Agarwal 2002, Saalu 2010). Mitochondrial membranes are particularly susceptible to ROS attack due to mitochondria being one of the primary producers of ROS (Zorov *et al.* 2014), the main instigator of membrane lipid peroxidation (Aitken 1995). A derivative of lipid peroxidation is 4-hydroxynonenal (4-HNE); it is one of the most researched products of lipid metabolism due to its reactivity and cytotoxicity (Spickett 2013). Once produced, 4-HNE can form adducts, modifying the functions of multiple targets within the sperm proteome such as α-tubulin (Baker *et al.* 2015), heat shock proteins (HSP; Bromfield *et al.* 2017) and succinate dehydrogenase (SDHA; Aitken *et al.* 2012*b*). Modification of SDHA by 4-HNE adduction dramatically increases mitochondrial superoxide production and causes a loss of the mitochondrial membrane potential (MMP; Aitken *et al.* 2012*b*). The loss of MMP also results in a decrease in the generation of ATP from mitochondrial respiration, leading eventually to cell death (Gaschler & Stockwell 2017, Uribe *et al.* 2017).

Dietary Membrane Lipid Replacement (MLR) supplements (NTFactor^®^ Lipids) are a novel therapeutic tool that allows damaged glycerophospholipids (GPL) in biological membranes to be replaced with exogenously supplied, undamaged GPL (Nicolson 2005, 2010, 2014, 2016, Nicolson & Ellithorpe 2006, Nicolson & Ash 2014). These supplements contain a mixture of the most common glycerophospholipids found within cell membranes, as well as unsaturated fatty acids (Nicolson & Ash 2017). Somatic cell studies have shown that MLR therapy allows peroxidised lipids in both the plasma and mitochondrial membranes to be replaced with exogenous GPL when somatic cells are exposed to an oxidative stress challenge (Nicolson & Ellithorpe 2006, Nicolson 2007, Nicolson & Conklin 2008). Interestingly, this phenomenon has also been demonstrated using human spermatozoa; GPL supplementation *in vitro* improved MMP, restored cellular functions and increased resistance to oxidative stress (Ferreira *et al.* 2018). Despite these promising *in vitro* results, there is an absence of experimental evidence showing the effects of MLR therapy in conjunction with artificial reproduction technologies such as sperm storage. Sperm storage technologies are a vital aspect of all equine artificial insemination programmes, and these storage methods can exert stressors that are quite detrimental to spermatozoa (Brinsko *et al.* 2000*a*,*b*, Batellier *et al.* 2001, Aurich 2005). It was hypothesised that the addition of sub-µm-sized glycerophospholipids to stallion spermatozoa during an *in vitro* oxidative challenge and during *in vitro* storage would provide protection against oxidative damage. As such, the overarching aim of this investigation was to investigate whether stallion spermatozoa are able to undergo membrane lipid replacement in the presence of physiological oxidative stressors, and if so, to ascertain whether the post-storage quality of stallion spermatozoa could be improved by *in vitro* GPL supplementation.

## Materials and methods

### Materials

Unless otherwise stated, all chemicals were purchased from Sigma-Aldrich (Australia). A modified Biggers, Whitten and Whittingham medium (BWW) a synthetic oviductal fluid (Biggers *et al.* 1971); containing 95 mM NaCl, 4.7 mM KCl, 1.7 mM CaCl_2_•2H_2_O, 1.2 mM KH_2_PO_4_, 1.2 mM MgSO_4_•7H_2_O, 25 mM NaHCO_3_, 5.6 mM d-glucose, 275 µM C_3_H_3_NaO_3_, 3.7 µL/mL 60% NaC_3_H_5_O_3_ syrup, 50 U/mL penicillin, 50 µg/mL streptomycin, 20 mM HEPES and 0.1% (w/v) polyvinyl alcohol, with an osmolarity of approximately 310 mOsm/kg and a pH of 7.4 was utilized throughout this study.

### Semen collection and preparation

Institutional and New South Wales State Government ethical approval was secured for the use of animal material in this study. All experiments were based on multiple ejaculates from five normozoospermic Shetland and miniature crossbred pony stallions (between 3 and 15 years of age) of proven fertility, held on institutionally approved premises. The stallions had access to native pasture 24 h a day and were supplementary fed with grass and lucerne hay once daily. Semen was collected using a pony-sized Missouri artificial vagina (AV; Minitube, Ballarat, VIC, Australia) with an in-line semen filter. The ejaculate was immediately diluted (2:1; extender:semen) with EquiPlus semen extender (Minitube, Ballarat, VIC, Australia).

Equipment and extender were maintained at temperatures between 30 and 37°C for the duration of semen collection and dilution. The tubes of extended semen were then transported to the laboratory in a polystyrene box at room temperature (RT; approximately 20–25°C). Once stallion semen samples were delivered to the laboratory, a maximum of 6 mL of extended semen was overlayed onto 3 mL of EquiPure (colloid gradient; Tek-Event Pty Ltd, Australia) and centrifuged at 400 ***g*** for 20 min. After removing the supernatant, the pelleted cells were resuspended in 1 mL of BWW solution for determination of sperm concentration.

Sperm concentrations were determined using a NucleoCounter NC-100™ (ChemoMetec, Denmark). Samples were then diluted out to approximately 20 to 25 × 10^6^ sperm/mL in BWW solution (unless otherwise stated). Washed spermatozoa were stored under aerobic conditions for all subsequent procedures.

### Preparation of sub-micrometre-sized glycerophospholipid micelles

Nutritional Therapeutics, Inc. (Hauppuage, NY, USA) specifically prepared the formulation of NTFactor^®^ Lipids for use in this study, which omitted the fructooligosaccharides from the formulation that are contained within the traditional dietary supplement to protect the GPL against degradation within the digestive system. GPL micelle stock solutions were prepared by adding 3% (w/v) modified NTFactor^®^ Lipids (Nutritional Therapeutics, Inc. of Hauppuage, NY, USA) to BWW. For micelle formation; ethanol (0.1% final volume) was added to the mixture, which was then vortexed until visibly dissolved. The GPL mixture was then placed in an ice bath and ultrasonicated at 20 kHz intermittently for 20 min (5 s on, 5 s off) using a probe sonicator (Bandelin Sonoplus, Germany). The resulting product was filtered through a 0.2 µm sterile syringe filter to isolate sub-micrometre-sized micelles as previously described (Ferreira *et al.* 2018).

### Evaluation of sperm motility

Computer assisted sperm analysis (CASA; IVOS, Hamilton Thorne, Danvers, MA) was used to objectively assess the motility parameters of sperm cells. The following settings were used; negative phase-contrast optics, recording rate of 60 frames/s, minimum cell size of 5 µm^2^, maximum cell size of 50 µm^2^, progressive average path velocity (VAP) threshold of 50 µm/s, slow (static) cell VAP threshold of 20 µm/s, slow (static) cell velocity (VSL) threshold of 0 µm/s, and threshold straightness (STR) of 75%. Cells exhibiting a VAP of ≥ 50 μm/s and an STR of ≥80% were considered progressive. A total of 3 µL of sperm from each sample was loaded into one chamber of a 20 µm deep four-chambered slide (Leja; Gytech Pty Ltd, Australia) with a stage temperature of 37°C. At minimum, 200 cells and 5 fields were analysed per sample.

### Flow cytometric analysis

All flow cytometry was performed using a FACSCanto II flow cytometer (Becton Dickinson, CA) with a 488-nm solid state laser. Emission measurements were made using 530/30 nm band pass (green/FITC), 585/42 nm band pass (red/PE), >670 nm long pass (far red/PerCp) and 780/60 nm band pass (far red/PECy7) filters. Using the forward scatter/side scatter dot plot, a gate was drawn and placed around the sperm population, excluding debris from the analysis. A minimum of 10,000 cells were analysed from each sample using FACSDiva V8.01 software (Becton Dickinson, CA).

### Flow cytometric measurement of mitochondrial ROS

The MitoSOX™ Red (MSR) reagent is membrane permeable and by virtue of its charge can specifically target the mitochondria. Under conditions of high mitochondrial ROS, the reagent is oxidized by superoxide and becomes highly fluorescent, allowing for a direct measurement of mitochondrial superoxide production. To run the MSR assay, 100 µL of sperm suspension was incubated for 15 min at 37°C with 2 µM MSR stain (Molecular Probes) and 5 nM SYTOX™ Green (Molecular Probes) viability stain. Staining controls included a positive dead control: 100 µL of spermatozoa was snap-frozen in liquid nitrogen and incubated for 15 min at 37°C with 5 nM SYTOX™ Green viability stain only; and an MSR positive control: 100 µL of sperm was incubated for 15 min with 100 µM arachidonic acid (AA) and 2 µM MSR stain only. The spermatozoa were then centrifuged at 500 ***g*** for 3 min; the supernatant was removed, and pellets were resuspended in 300 µL BWW for analysis by FACSCanto II. As all dead cells are read positive for MSR (due to an artefact associated with the ethidium-based fluorophore), only live-cell data were collected for statistical analyses.

### Flow cytometric measurement of cellular 4-hydroxynonenal adduction

4-Hydroxynonenal (4-HNE) is a well-characterized derivative of lipid peroxidation and is a stable marker for measuring levels of oxidative stress. To measure 4-HNE levels in spermatozoa, 100 µL of sperm were centrifuged at 500 ***g*** for 3 min, the supernatant was snap-frozen in liquid nitrogen for use in subsequent experiments, and sperm pellets were resuspended in 50 µL of BWW containing 0.02 µg/µL (1:50 of antibody as supplied) rabbit α-HNE primary antibody (Abcam) and 1:2000 (v/v) LIVE/DEAD™ Fixable Far Red Dead Cell stain (Molecular Probes). Samples were incubated at 37°C for 30 min. Spermatozoa were then centrifuged at 500 ***g*** for 3 min; the supernatant was removed, and pellets resuspended in 100 µL BWW containing 2 µg/µL goat α-rabbit IgG peroxidase conjugate (Alexa Fluor 488 goat α-rabbit secondary antibody) and incubated at 37°C for 15 min. Spermatozoa were centrifuged at 500 ***g*** for 3 min, supernatant was removed, and pellets were washed a second time to remove all unbound antibody. Spermatozoa were then resuspended in 300 µL BWW and placed into clean FACS tubes for analysis by FACSCanto II. To allow for specific gating, staining controls were used. These included; snap-frozen LIVE/DEAD™ positive control, where 100 µL of spermatozoa was snap-frozen in liquid nitrogen, thawed, and incubated for 30 min at 37°C with 1:2000 (v/v) LIVE/DEAD™ Fixable Far Red Dead Cell stain only, and a secondary antibody (Alexa Fluor 488 goat α-rabbit secondary antibody) only control.

Additionally, spermatozoa were incubated with 50 µM AA with and without the addition of GPL for 2 h at 37°C. The staining protocols for flow cytometric measurement of cellular 4-HNE adduction were followed, as described above, with some modifications; the LIVE/DEAD™ stain was substituted with 7.5 µM propidium iodide (PI; added immediately before assessment). Spermatozoa were mounted on slides with paraformaldehyde (0.01%, v/v) to suppress motility. Representative images of spermatozoa were taken with fluorescent microscopy.

### Measurement of 4-hydroxynonenal within the spent media

Concentration of 4-HNE adducts within the snap-frozen supernatant (spent media) collected as outlined in ‘Flow cytometric measurement of cellular 4-hydroxynonenal adduction’ section, was analysed with the Lipid Peroxidation (4-HNE) ELISA Kit (Abcam) as per the manufacturer’s instructions.

### Statistical analyses

All statistical analyses were conducted using JMP Pro, version 14.2.0 (SAS Institute, Cary, NC). A Student’s *t*-test and was used when comparing two treatments to determine if the observed changes that occurred with treatments were greater than what could be probable by chance. For 17°C storage experiments, a REML analysis was used to compare repeated measures over time. For this model, stallion name was selected as a random effect, and comparisons were made within time points; comparison of group mean values with the corresponding controls was achieved using Dunnett’s test. These parametric methods were only used if the data distribution was normal according to the Anderson–Darling goodness-of-fit test, while the assumed homogeneity of variances was checked using the Bartlett test. Where the data were not normally distributed, the non-parametric Wilcoxon signed-rank test was used. The specific tests used for each section of this study are clearly indicated within the figure legends. Significance was determined when *P* ≤ 0.05. All data are displayed as means ± s.e.m.

## Experimental design

### Experiment 1: Incubation of stallion spermatozoa with glycerophospholipids in the presence of induced oxidative stress

Stallion spermatozoa were exposed to increasing concentrations of AA (0, 25, 50 and 100 µM; concentrations which have been used in previous studies, and reflects physiological AA concentrations within cells; Moazamian *et al.* 2015, Tallima & El Ridi 2018) to induce oxidative stress, with and without the addition of GPL at a final concentration of 0.1% (v/v; a concentration which has previously been optimized for use with human spermatozoa; Ferreira *et al.* 2018). Samples were incubated at 37°C for 1 h (*n* = 9 ejaculates), then analysed for motility (‘Evaluation of sperm motility’ section), viability (SYTOX™ green; ‘Flow-cytometric measurement of mitochondrial ROS’ section) and mitochondrial ROS (MSR; ‘Flow cytometric measurement of mitochondrial ROS’ section). To avoid interference from large GPL micelles in CASA motility measurements, all samples were washed via centrifugation after treatment at 500 ***g*** for 3 min, resuspended in RT BWW and immediately analysed for motility with no further incubation. Additionally, 4-HNE cellular adduction (‘Flow-cytometric measurement of cellular 4-hydroxynonenal adduction’ section) and free 4-HNE in the spent medium (‘Measurement of 4-hydroxynonenal within the spent media’ section) were measured following GPL supplementation in the presence of an oxidative challenge using 50 µM AA.

### Experiment 2: Addition of ultrasonicated glycerophospholipids to stallion sperm storage media to aid in resistance to oxidative stress

To ascertain whether exogenous GPL in sperm storage media can improve the quality of spermatozoa (*n* = 9 ejaculates), stallion spermatozoa were resuspended in BWW with and without the addition of GPL (0.1% v/v) and incubated at 17°C for 72 h. Motility (‘Evaluation of sperm motility’ section) and viability (SYTOX™ green; ‘Flow cytometric measurement of mitochondrial ROS’ section) were analysed every 24 h (24, 48 and 72 h). To avoid interference from large GPL micelles in CASA motility measurements, all samples were centrifuged after treatment at 500 ***g*** for 3 min, resuspended in RT BWW and immediately analysed for motility with no further incubation.

## Results

### Experiment 1: Incubation of stallion spermatozoa with glycerophospholipids in the presence of induced oxidative stress

**Figure 1:**
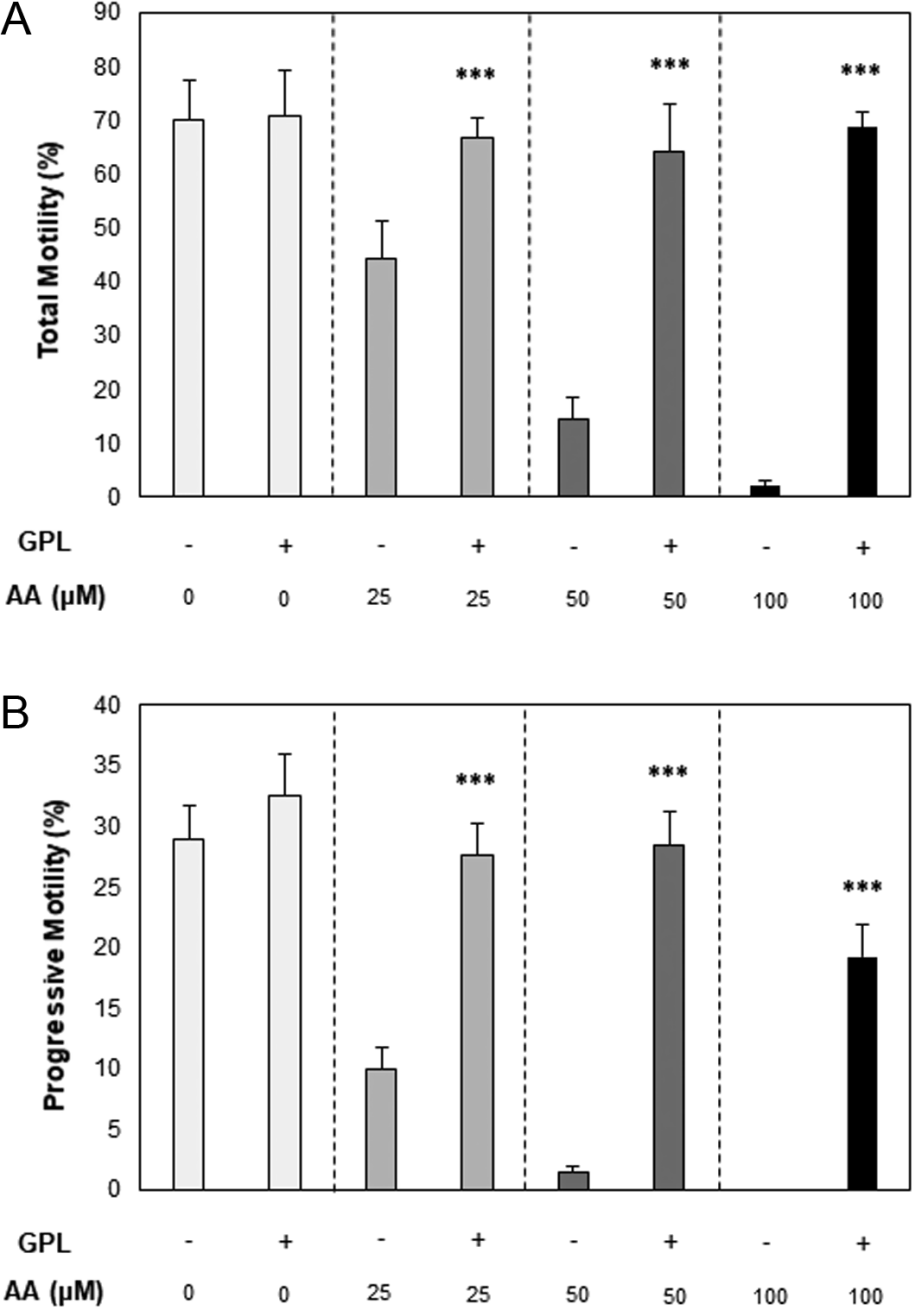
Dose–response curves of total and progressive motility of stallion spermatozoa vs arachidonic acid (AA) concentration. (A) Total motility and (B) progressive motility of stallion spermatozoa, obtained after incubation in the presence of increasing concentrations of arachidonic acid only (AA; −) or in the presence of increasing concentrations of arachidonic acid with the addition of GPL (+). All data are displayed as means ± s.e.m. (***P ≤ 0.001; Student’s *t*-test was used for 0, 25 and 50 µM AA data; Wilcoxon signed rank test was used for 100 µM AA data).

When GPL were available to stallion spermatozoa during an AA-induced oxidative challenge, supplemented spermatozoa had higher total motility at 25, 50 and 100 µM AA doses (44.2 ± 7.2 vs 66.7 ± 3.8%, 14.4 ± 4.1 vs 64.4 ± 8.7% and 2 ± 1 vs 68.8 ± 2.9%, respectively; all *P* ≤ 0.001; Fig. 1A). The GPL-supplemented spermatozoa also displayed improved progressive motility at 25, 50 and 100 µM AA doses (9.9 ± 1.7 vs 27.7 ± 2.6%, 1.4 ± 0.6 vs 28.4 ± 2.8% and 0 ± 0 vs 9.3 ± 2.6%, respectively; all *P* ≤ 0.001; Fig. 1B). It was also noted that GPL-supplemented spermatozoa were able to maintain the same level of total motility across all AA doses (Fig. 1A; overall mean of 67.7 ± 1.5%; *P* > 0.05).

Additional sperm motility parameters were analysed throughout the oxidative challenge, demonstrating that when GPL were available to stallion spermatozoa, straight line velocity (VSL; 40.4 ± 5.3 vs 70.6 ± 4.9 µm/s, 21.6 ± 3.8 vs 69.2 ± 5.8 µm/s and 9.5 ± 2.1 vs 50.9 ± 4.1 µm/s) curvilinear velocity (VCL; 143.4 ± 13 vs 204.6 ± 3.6 µm/s, 82.1 ± 7.5 vs 193 ± 6.6 µm/s and 40.8 ± 10 vs 160.7 ± 7.8 µm/s) and average path velocity (VAP; 69.5 ± 10 vs 113.9 ± 3.7 µm/s, 31.3 ± 5.2 vs 104.3 ± 5.1 µm/s and 13.4 ± 2.9 vs 81.9 ± 5.9 µm/s) all improved (*P* ≤ 0.001) compared to the non-supplemented spermatozoa at 25, 50 and 100 µM AA doses, respectively.

**Figure 2:**
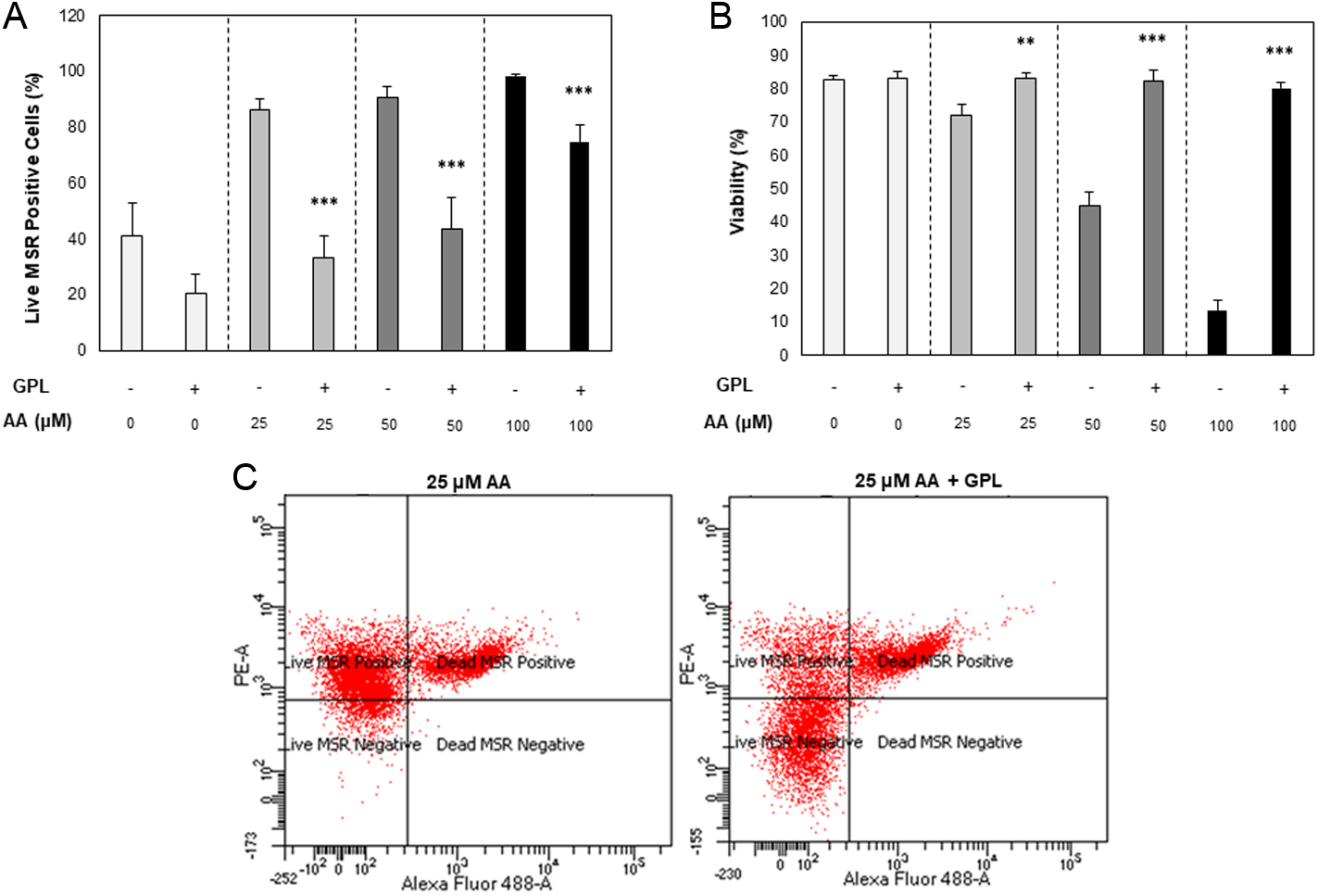
Flow cytometric analysis of mitochondrial ROS within stallion spermatozoa. (A) Mitochondrial ROS levels measured by MitoSOX™ Red reagent (MSR), (B) Total cell viability measured with SYTOX™ Green Dead Cell Stain obtained after co-incubation in the presence of increasing concentrations of arachidonic acid only (AA; −) or in the presence of increasing concentrations of arachidonic acid with the addition of GPL (+), and (C) a representative flow cytometric dot plot depicting the fluorescent changes in spermatozoa after incubation with 25 µM AA (left) and 25 µM AA with the addition of GPL (right). All data are displayed as means ± s.e.m. (*P ≤ 0.05, **P ≤ 0.01, ***P ≤ 0.001; Student’s *t*-test).

To determine whether the improved motility in the presence of GPL (Fig. 1) was due to MLR and ROS amelioration, a flow cytometric MSR assay was conducted. When GPL were present in the incubation media, mitochondrial ROS levels were reduced at 25, 50 and 100 µM AA doses (86 ± 4 vs 33.2 ± 8%, 9.7 ± 3.8 vs 43.8 ± 11.1% and 98.2 ± 0.6 vs 74.8 ± 6.11%, respectively; *P* ≤ 0.001; Fig. 2A). Furthermore, spermatozoa supplemented with GPL showed no loss of viability at all AA concentrations assessed (overall mean of 82.2 ± 0.7%, *P* > 0.05) whereas the non-supplemented spermatozoa showed a reduction in viability at 25, 50 and 100 µM AA doses (71.9 ± 3.4 vs 83.2 ± 1.7%, *P* ≤ 0.01; 44.9 ± 4.3 vs 82.4 ± 3%, *P* ≤ 0.001; and 13.5 ± 2.9 vs 80.2 ± 1.6%, *P* ≤ 0.001 respectively; Fig. 2B).

**Figure 3:**
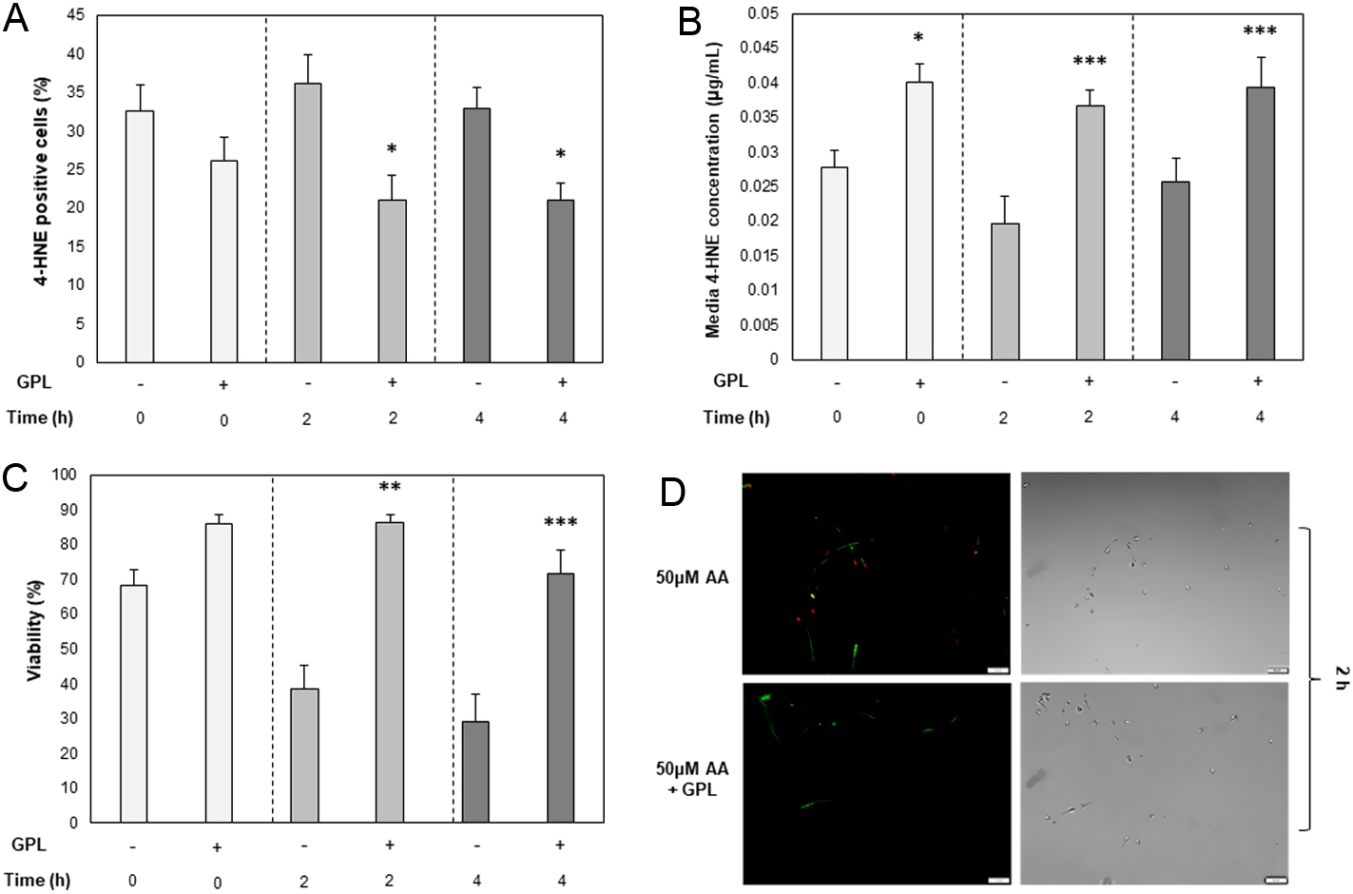
Analysis of 4-HNE adduction. (A) Total cellular 4-HNE adduction, utilizing α-HNE primary and Alexa Fluor 488 secondary antibodies, (B) Concentration of 4-HNE adducts within the spent sperm storage media and, (C) Total cell viability measured with LIVE/DEAD™ fixable far red dead cell stain obtained after co-incubation in the presence of 50 µM arachidonic acid only (AA; −) or in the presence of 50 µM AA with the addition of GPL (+), measured at regular 2 h intervals over a total of 4 h. (D) Fluorescent and phase images of spermatozoa depicting 4-HNE adduction (green) utilizing α-HNE primary and Alexa Fluor 488 secondary antibodies, after 2 h incubation with 25 µM AA only (top) and 25 µM AA with the addition of GPL (Bottom). Red fluorescence (PI) indicates a dead cell. Original magnification ×400. All data are displayed as means ± s.e.m. (*P ≤ 0.05, **P ≤ 0.01, ***P ≤ 0.001; Student’s *t*-test).

To analyse the impact of time on lipid peroxidation; stallion spermatozoa were incubated with or without GPL in the presence of 50 µM AA only. Cellular 4-HNE adduction, free 4-HNE adducts within the spent media, and viability were measured at 0, 2 and 4 h of incubation at 37°C. The addition of GPL to the incubation media resulted fewer 4-HNE positive cells at 2 and 4 h (36.1 ± 3.9 vs 20.9 ± 3.3%, 32.9 ± 2.7 vs 20.9 ± 2.3%, respectively; *P* ≤ 0.05; Fig. 3A), and a higher concentration of 4-HNE adducts within the spent media at 0, 2 and 4 h (0.028 ± 0.003 vs 0.04 ± 0.003 µg/mL, *P* ≤ 0.05; 0.02 ± 0.004 vs 0.037 ± 0.003 µg/mL, *P* ≤ 0.001; 0.026 ± 0.003 vs 0.039 ± 0.004 µg/mL, *P* ≤ 0.001, respectively; Fig. 3B). The addition of GPL to the incubation media also resulted in higher viability at 2 and 4 h time points compared to the non-supplemented treatment (38.5 ± 6.9 vs 86.4 ± 2.2%, *P* ≤ 0.001; and 29.1 ± 8 vs 71.7 ± 6.7%, *P* ≤ 0.001, respectively; Fig. 3C).

### Experiment 2: Addition of glycerophospholipids to stallion sperm storage media to aid in resistance to oxidative stress

**Figure 4:**
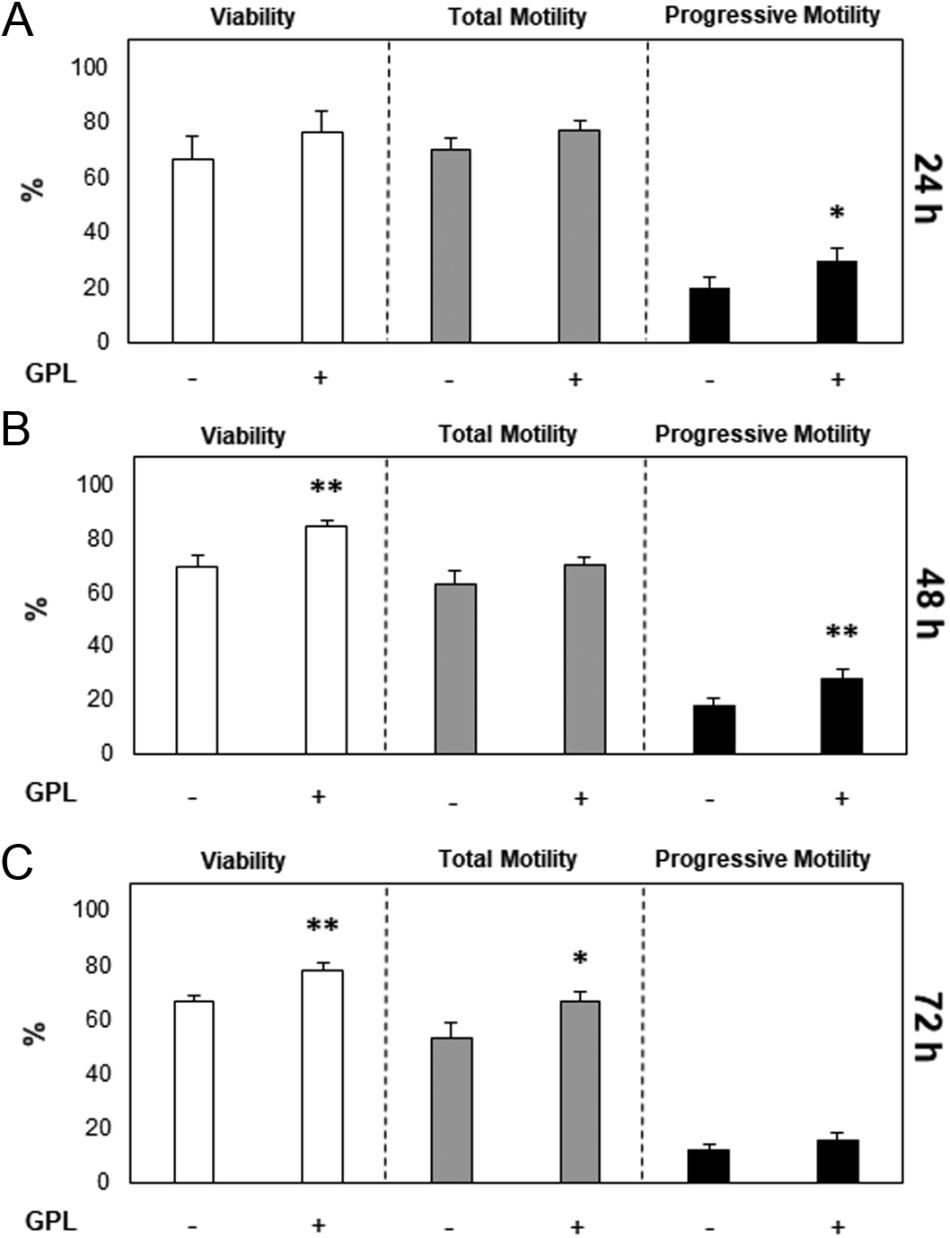
Sperm were stored at 17°C with the addition of GPL. Sperm were stored at 17°C in BWW only (−) or BWW with the addition of GPL (+). Total cell viability measured with SYTOX™ Green Dead Cell Stain and sperm motility parameters were measured at (A) 24 h, (B) 48 h, and (C) 72 h. All data are displayed as means ± s.e.m. (*P ≤ 0.05. **P ≤ 0.01; REML analysis).

To ascertain whether the addition of GPL to sperm storage media could improve post-storage sperm quality via enhanced resistance to oxidative stress *in vitro*, stallion spermatozoa were stored over 72 h with and without GPL at 17°C. Storage at 17°C has previously been found optimal for non-cooled sperm storage by our laboratory, sperm metabolism is not curtailed at this temperature, and therefore, spermatozoa suffer the consequences of mitochondrial ROS production (Gibb *et al.* 2018). When GPL were added to sperm storage media, an improvement in progressive motility was observed after 24 h (19.9 ± 3.8 vs 29.3 ± 5.11; *P* ≤ 0.05). An improvement in sperm viability was observed after 48 h, as well as an improvement in progressive motility (69.5 ± 4.2 vs 84.2 ± 2.2%, *P* ≤ 0.01; and 18.2 ± 2.6 vs 27.9 ± 3.8%, *P* ≤ 0.01, respectively; Fig. 4B). Likewise, after 72 h of sperm storage there was an improvement in sperm viability and total motility compared to the non-supplemented group (66.4 ± 2.6 vs 78.1 ± 2.9%, *P* ≤ 0.01; and 53 ± 5.6 vs 66.3 ± 3.5%, *P* ≤ 0.05, respectively; Fig. 4C).

## Discussion

This is the first study to confirm that stallion spermatozoa are capable of replacing oxidized membrane GPL for exogenously supplied GPL to repair cellular membranes, restore sperm functions and protect against oxidative stress *in vitro*. This study supports previously published findings in which membrane lipid replacement therapy utilizing NTFactor^®^ Lipids restored mitochondrial function *in vivo*; reducing fatigue in aged patients and patients suffering from a variety of fatigue-related clinical diagnoses, such as fibromyalgia, Lyme’s disease, and chronic fatigue syndrome (Nicolson & Ellithorpe 2006, Nicolson 2014, Nicolson & Ash 2014), while increasing mitochondrial function (Agadjanyan *et al.* 2003, Nicolson 2014). NTFactor^®^ Lipids are a commercially available mixture of the most common glycerophospholipids found within cell membranes; as well as unsaturated fatty acids, and this natural supplement has previously been shown to provide resistance to oxidizing agents such as hydrogen peroxide (H_2_O_2_), improving viability and motility of human spermatozoa *in vitro* (Ferreira *et al.* 2018).

Oxidative damage in stallion spermatozoa was stimulated via co-incubation with varying doses of AA (Hong *et al.* 1986), promoting degradation of cell membranes by producing toxic lipid aldehydes, causing cell damage and dysregulating cellular functions (Aitken *et al.* 2012*b*). At this point, we sought to clarify that AA was not one of the PUFA contained in NTFactor^®^ Lipids (Nicolson & Ash 2017), the commercial preparation of the GPL utilized in this study and would not confound the results. Published compositions of NTFactor^®^ Lipids did not reveal the presence of AA (Nicolson 2016). A previous study by Ferreira *et al.* (2018) utilized H_2_O_2_ to induce oxidative stress in human spermatozoa. In this situation, supplementation with fluorescently labelled GPL allowed visualization of incorporated lipids to ensure that lipid membrane exchange had occurred. As H_2_O_2_ can be directly scavenged by GPL, for this study, we selected AA as an alternative oxidative stressor, as we wished to ensure that the effects we were observing were not due to the direct sequestering of H_2_O_2_ by GPL, as opposed to the effects of MLR.

Flow cytometric analysis confirmed that 4-HNE adduction was significantly diminished when GPL micelles were made available to oxidatively stressed spermatozoa (Fig. 3A). Additionally, the significant reduction of mitochondrial ROS production observed in this study (Fig. 2A) could be attributed to the reduction in the downstream effects of 4-HNE adduction; 4-HNE exacerbates mitochondrial superoxide production by preferential adduction to SDHA, perturbing mitochondrial function and driving ROS production (Ball *et al.* 2001, Aitken *et al.* 2012*b*). This increase in ROS can further peroxidise additional membrane lipids, increasing the production of 4-HNE, and as a consequence, this can further exacerbate the stress cycle (Aitken *et al.* 2012*b*). The ability of spermatozoa to substitute oxidized GPL from their plasma and mitochondrial membranes with fresh GPL from the extracellular milieu has previously been demonstrated (Ferreira *et al.* 2018).

When peroxidation of membrane phospholipid PUFAs occurs, active removal or repair processes are initiated. One such process involves the cleavage of the damaged PUFA at the site of peroxidation by phospholipase A2 (PLA_2_) enzymes, which play an intrinsic role in protecting cell membranes from oxidative damage (Sevanian & Kim 1985). This process has been shown to be present and active in spermatozoa (Collodel *et al.* 2020). Indeed, PLA_2_ activity is highly correlated with the degree of lipid peroxidation in membranes (Sevanian *et al.* 1983), and if not replaced by lipids from exogenous sources, PLA_2_ activity may be sufficient to perturb cellular membranes to the point of cell lysis via the creation of lysophosphlipids (Shier 1979). Exogenously supplied GPL, such as those used in the present study, may be transported across membranes by binding to transmembrane phospholipid-translocase proteins (ATP-independent biogenic flippases), which have recently been characterized in the outer intracellular membranes of bovine spermatozoa (Rajasekharan *et al.* 2013). GPL may also be taken up by most cells as small liposomes and lipid globules that enter the cell by endocytosis (Nicolson 2014, Nicolson & Ash 2017). Organelles such as mitochondria are able to transfer damaged membrane lipids via direct contact, as well as via lipid transport proteins that shuttle membrane phospholipids between inner and outer membranes (Chatterjee & Gagnon 2001). Once they arrive at the site of a damaged membrane, GPL can then be then enzymatically modified to replace damaged lipids (Nicolson & Ash 2017). The lower levels of 4-HNE cellular adduction (Fig. 4A) and increased 4-HNE concentration within the spent media (Fig. 4B) observed following GPL supplementation lends support to the hypothesis that this membrane lipid replacement mechanism is indeed functional in stallion spermatozoa, as GPL supplementation inhibited the downstream effects of lipid peroxidation before reactive aldehydes such as 4-HNE were produced and the subsequent mitochondrial ROS increase was able to decrease cell viability (Figs 3A and 4C; Aitken *et al.* 1989). Therefore, membrane lipid replacement must be occurring whilst still in the lipid hydroperoxide intermediate metabolite stage (such as 15-HPETE, which is then later degraded into 4-HNE within the media), but before being completely degraded to 4-HNE cellular adducts and propagating the oxidative stress cycle (Aitken *et al.* 2012*b*, Ayala *et al.* 2014).

The underlying mechanism by which membrane lipid replacement functions *in vitro* to modify spermatozoa are yet to be conclusively elucidated; at the time of this study, there was only one publication on the use of MLR to treat spermatozoa *in vitro* (Ferreira *et al.* 2018). The plasma membrane surrounding the sperm head displays differences in lipid compositions and therefore, differences in fluidities (Oresti *et al.* 2011). Membranes with these structural asymmetries have been shown to be in contact with intracellular membranes allowing the exchange of lipids through direct membrane–membrane contact (Wolfe *et al.* 1998). Whether this lipid exchanging mechanism is entirely passive or involves more complex components such as lipid transport proteins or fatty acid cleaving enzymes such as PLA_2_ is unclear and warrants further investigation.

The addition of GPL micelles to AA-treated spermatozoa significantly improved viability (Figs 2B and 3C), as well as a number of motility parameters (Fig. 1). This is most certainly a downstream effect of the amelioration of the aforementioned lipid peroxidation and oxidative stress cycle, as 4-HNE adduction to succinate dehydrogenase (complex II of the mitochondrial electron transport chain) perturbs ATP production and instigates the apoptotic cascade (Koppers *et al.* 2011). When this pathway is activated, mitochondrial ROS rapidly triggers a loss of transmembrane potential, followed by a loss of ATP production necessary for motility, caspase activation and membrane disorganization (phosphatidylserine translocation). This is followed by oxidative DNA damage and strand breakage, and ultimately cell death (Aitken *et al.* 2012*b*).

The observation of the protective effects of GPL during an oxidative stress challenge led to the notion that exogenous GPL micelles might be a beneficial additive to sperm storage media, ameliorating ROS and therefore improving the post-storage quality of stallion spermatozoa stored at 17°C. During conventional sperm storage (chilled and cryopreserved) the majority of damage to spermatozoa during occurs at the sperm plasma membrane when it transitions between a fluid (liquid crystalline) phase to a gel state during cooling and rewarming, resulting in irreversible mechanical damage (Sieme *et al.* 2015), which may not necessarily be oxidative in nature. This damage affects the functional and molecular state of the sperm membrane, resulting in a reduction of membrane fluidity (Aitken *et al.* 2006). Cooling-induced reduction in sperm motility and function has formerly been suspected to be a symptom of the formation of ROS and successive lipid peroxidation (Aitken 1995, Chatterjee & Gagnon 2001). However, the irreversible mechanical damage which the plasma membrane endures during this process may far outweigh the damage sustained from the formation of ROS. Previous studies have also established that unlike other species, such as humans (Surmon *et al.* 2009), antioxidant supplementation during cooled storage is of minimal benefit to stallion spermatozoa (Baumber *et al.* 2000, 2005). Therefore, this study focused on 17°C sperm storage, a temperature which this group has previously shown to be optimal for long-term storage of stallion spermatozoa (Gibb *et al.* 2018), but one at which lipid peroxidation is a recognized consequence of uninhibited sperm metabolism (Aitken *et al.* 2012*a*). Supplementation of stallion spermatozoa with GPL during 17°C *in vitro* storage successfully diminished the effects of lipid peroxidation, most certainly due to reasons stated previously, improving sperm viability and motility parameters post 24, 48 and 72 h storage (Fig. 4).

The authors acknowledge the limitations of the study, being that a conclusive mechanism was not established by the use of inhibitors, in the case of PLA_2_. These further experiments aim to be the main focus of future studies.

In conclusion, our results indicate that incubation of stallion spermatozoa with a mixture of sub-µm-sized GPL micelles at a concentration of 0.1% (Nicolson 2010), does indeed result in the incorporation of exogenous GPL into the sperm plasma and inner membranes as previously described in human spermatozoa by Ferreira *et al.* (2018). Due to this lipid substitution, incubation of stallion spermatozoa with GPL micelles successfully diminished lipid peroxidation, improving the viability and motility parameters of stallion spermatozoa during *in vitro* storage at 17°C. With this knowledge, the investigators first propose that glycerophospholipids may be a beneficial additive within commercial semen extender preparations for stallions and possibly other mammalian spermatozoa as well, and secondly, further exploration should be considered into possible benefits of utilizing GPL as an *in vivo* oral supplement for animals who exhibit poor fertility as a result of elevated sperm lipid peroxidation and oxidative DNA damage.

## Declaration of interest

The authors declare that there is no conflict of interest that could be perceived as prejudicing the impartiality of the research reported.

## Funding

This work was supported by Australian Research Council Linkage grant #LP160100824.

## Author contribution statement

A M conducted the experiments, statistical analysis and wrote the manuscript. R A provided guidance and assisted with manuscript editing. G N supplied NTFactor^®^ Lipids and assisted with manuscript editing. A R S aided with experiments and stallion semen collections. A S and G D provided guidance. Z G conceived and funded the study, assisted with stallion semen collections, and reviewed and edited the manuscript.
